# Free-style puzzle flap as a cross-leg pedicled flap: the concept of re-using a flap in acute burns, a case report

**DOI:** 10.1186/s41038-018-0107-2

**Published:** 2018-02-02

**Authors:** Kevin Serror, Marc Chaouat, Golda Romano, Magali Schmidt, Alice Blet, Maurice Mimoun, David Boccara

**Affiliations:** Department of Plastic Reconstructive Surgery, Burn Center, Hôpital Saint Louis, AP-HP 1 Avenue Claude Vellefaux, 75010 Paris, France

**Keywords:** Burns, Free flap, Pedicled flap, Puzzle flap, Cross-leg

## Abstract

**Background:**

In well-selected cases, flaps can play a pivotal role in optimizing outcomes in the acute phase of burns. A previous redundant flap could be reused or recycled as a donor site from which a new flap could be raised.

**Case presentation:**

We report the case of a patient with full thickness burns on both legs, leading to the exposure of joints of the right ankle and the right foot and left patellar tendon. The right lower extremity was covered with a free musculo-cutaneous latissimus dorsi flap. Then, a musculo-cutaneous cross-leg flap pedicled on the anterior branch and centered on a perforator was harvested from the previous redundant flap to cover the controlateral knee.

**Conclusion:**

Sequential flap coverage can be considered in cases of extensive soft tissue defects and particularly in burns. This case illustrates that re-using a redundant part of a previous flap to cover another defect is a safe and interesting alternative in the event of a lack of donor sites or to save donor sites for later reconstruction of contracted burn scars.

## Background

The mainstay of operative treatment in burns remains as split thickness skin autografts. Platt et al. reported that skin grafts represent more than 95% of procedures in acute burns [1]. Nevertheless, in well-selected cases, flaps can play a pivotal role in optimizing outcomes in the acute phase [2–6]. They allow the preservation of otherwise unsalvageable deep burns exposing joints, tendons, nerves or vessels. In cases of extensive soft tissue defects following the debridement of devitalized tissues, the surface or the location of the defects may exceed the possibility of coverage with a single flap. Havlik and Ariyan [7] reported cases of re-using a previous flap as a donor site. Mun [8] described a concept in which a previous redundant flap could be reused or recycled as a donor site from which a new flap could be raised. Feng et al. [9] called this concept a “free-style puzzle flap” and reported a series of oncologic and post-traumatic soft tissue defects covered with puzzle flaps.

Our aim in this article is to report the case of a patient in the acute phase of a burn requiring a free-style puzzle flap combined with a cross-leg pedicled flap.

## Case presentation

**Fig. 1:**
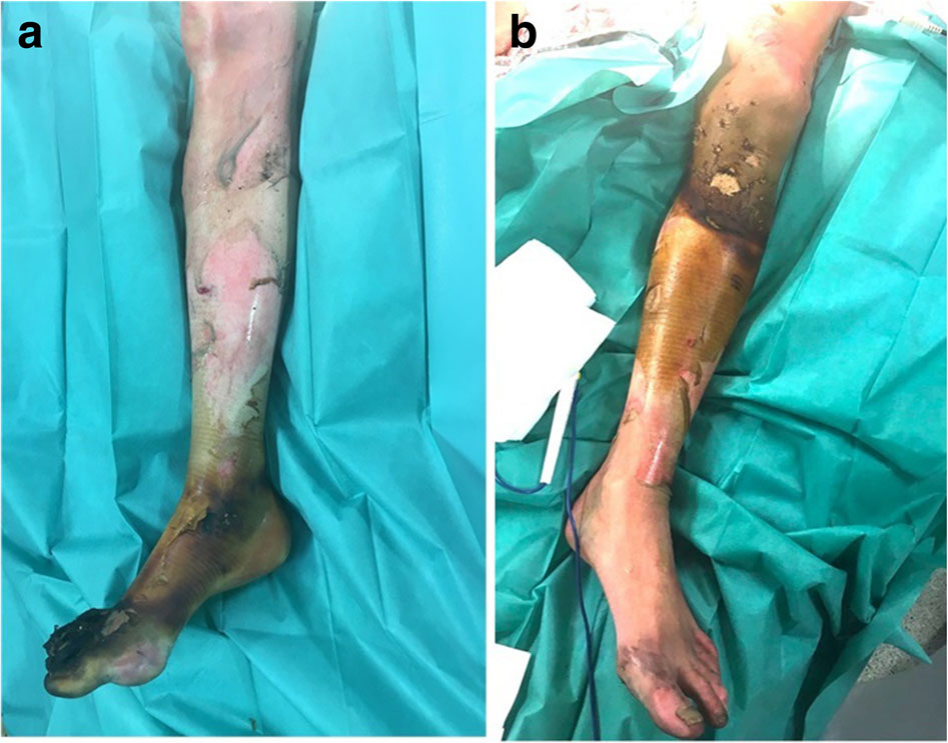
Appearance of a 55-year-old woman patient's right (**a**) and left (**b**) legs with full thickness burns at admission, before the releasing incisions were performed

**Fig. 2:**
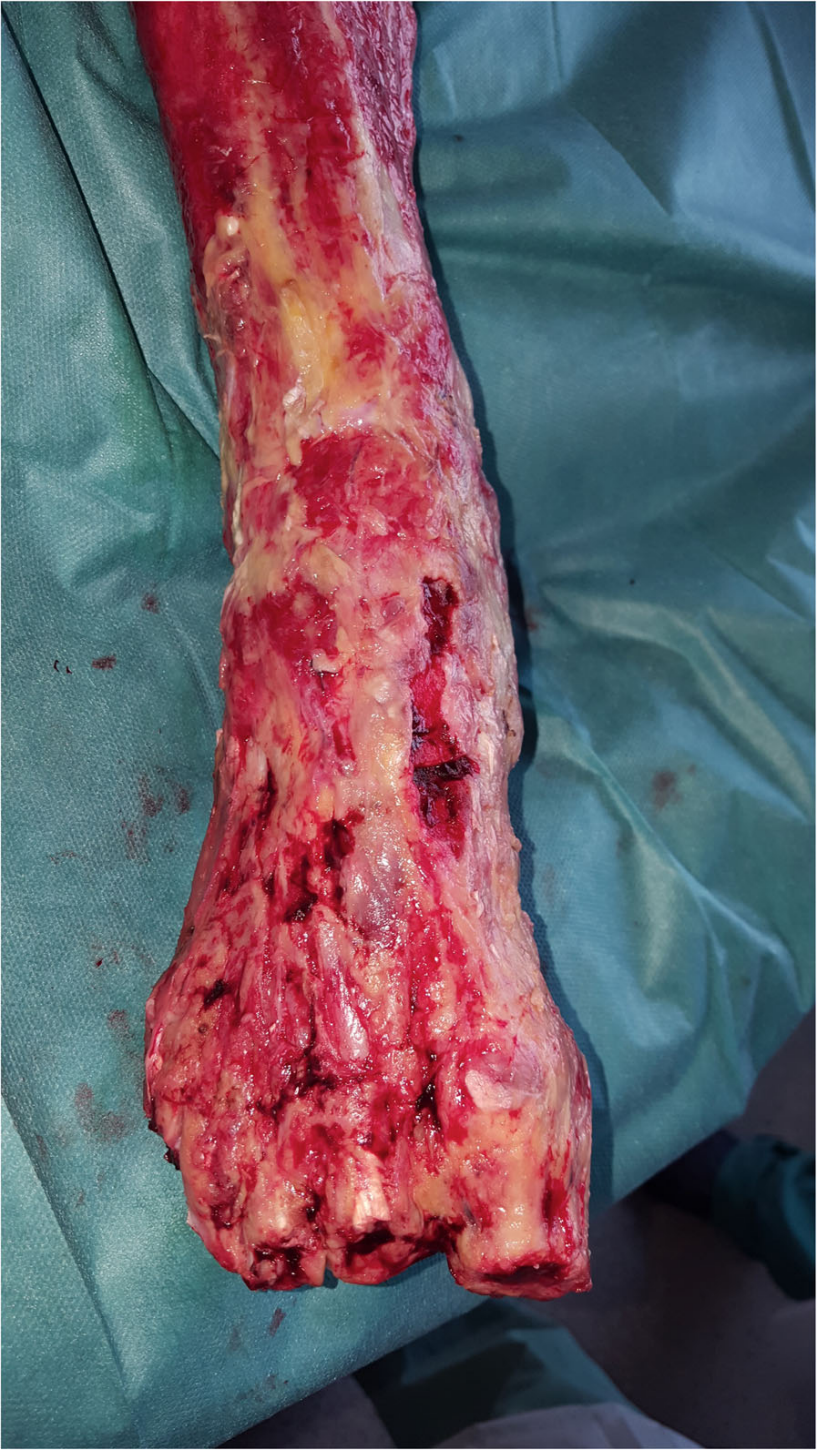
Appearence of lower right extremity of a 55-year-old woman burn patient after debridment of necrotic tissues. Tibia and fibula were exposed. Ankle, mediotarsal, and tarso-metatarsal capsules were burnt and some joints were open

**Fig. 3:**
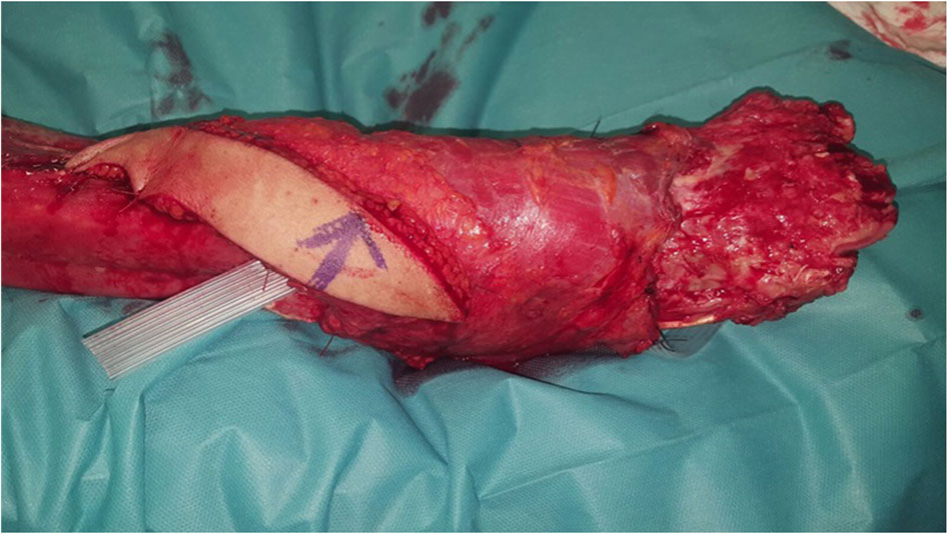
Appearence of lower right extremity of a 55-year-old woman burn patient after a free latissimus dorsi musculo-cutaneous flap with vertical skin paddle was performed to cover dorsal foot and ankle

**Fig. 4:**
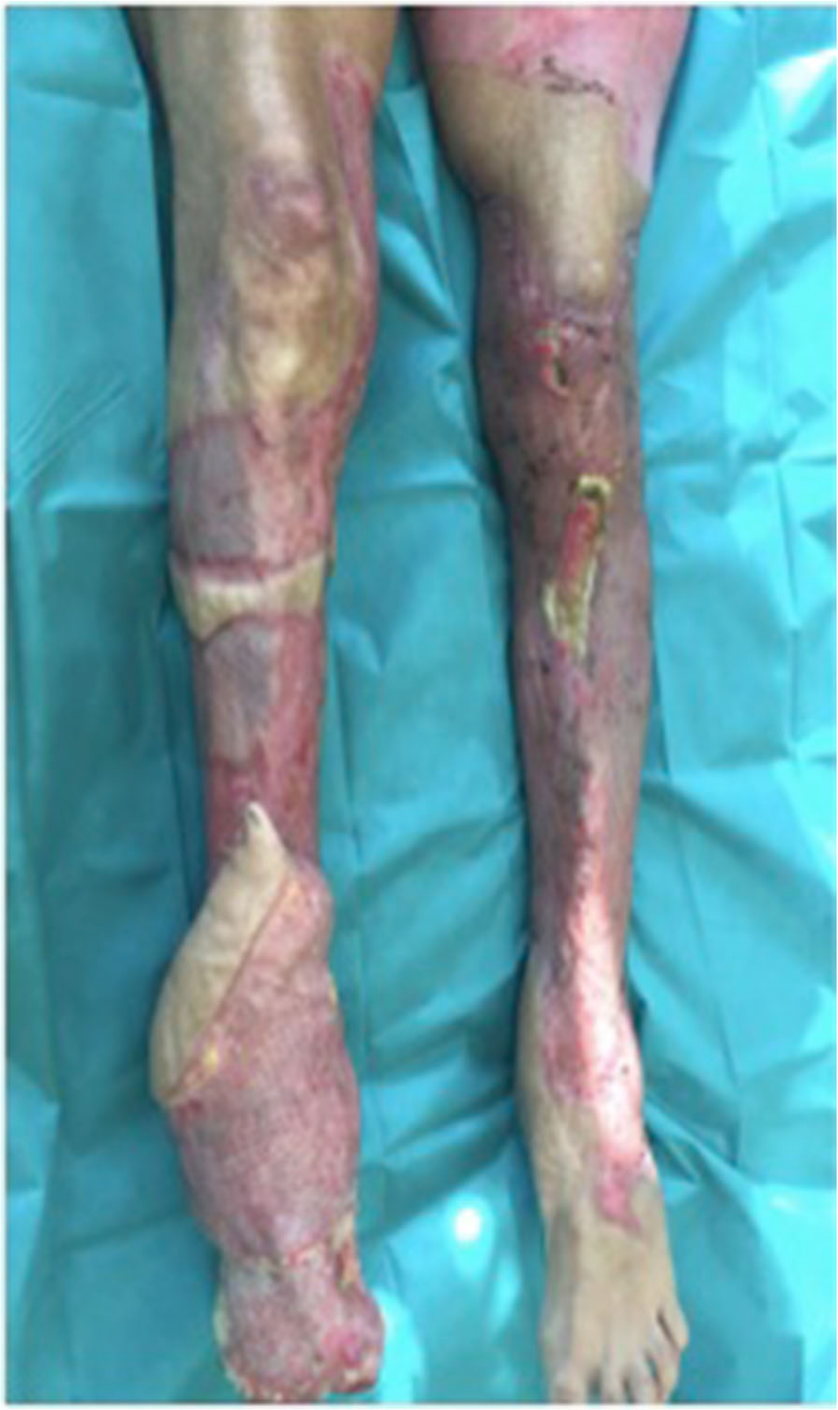
Appearence of lower limbs of a 55-year-old woman burn patient with exposure of left patellar tendon and superior third of the tibia requiring coverage with a flap

**Fig. 5:**
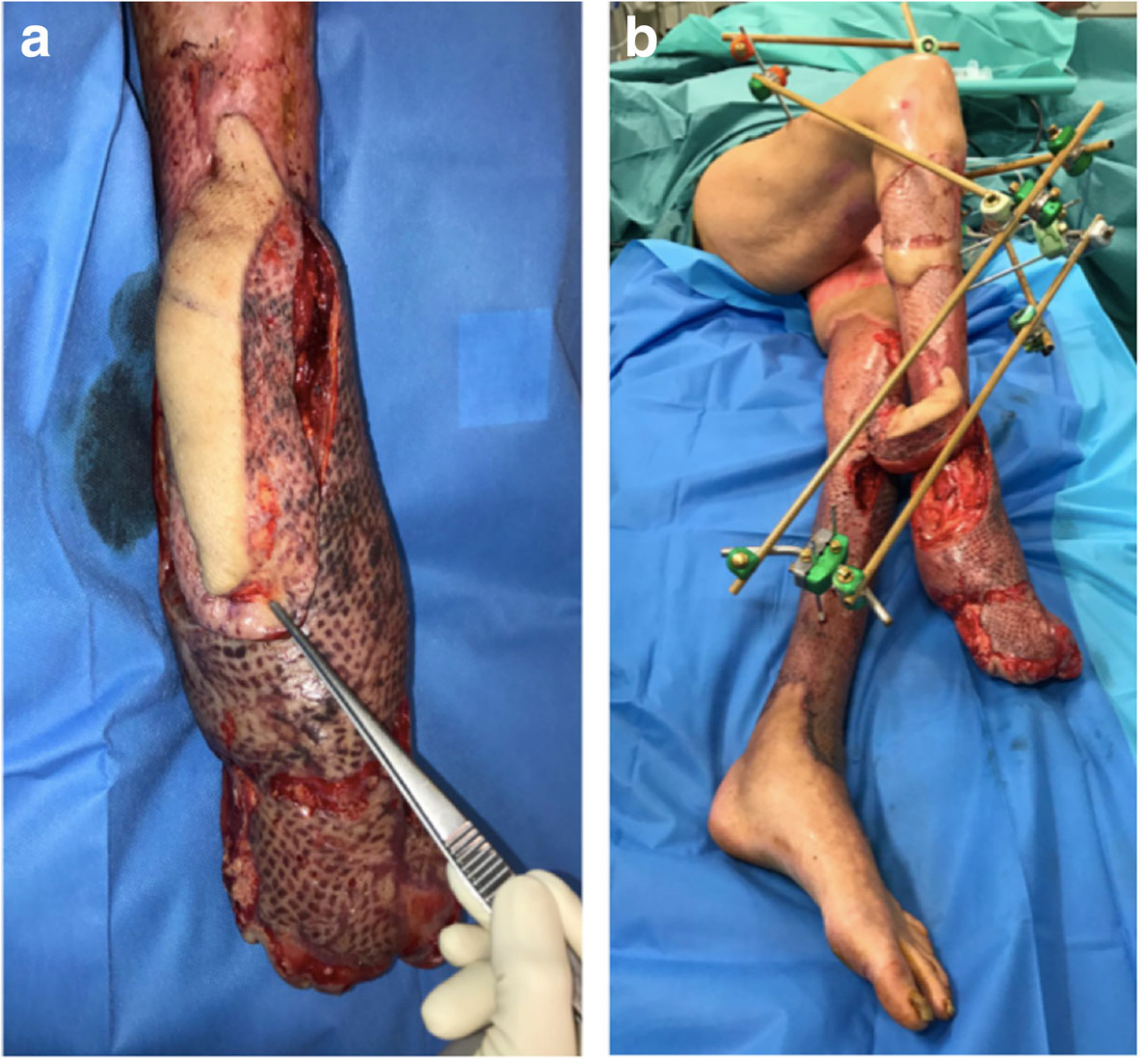
Appearance of both legs of a 55-year-old woman burn patient after a pedicled flap was raised from the previous free flap; (**a**) a cross leg transferred to cover the left patellar tendon and (**b**) both legs were immobilized together using an external fixation (Hoffmann apparatus)

**Fig. 6:**
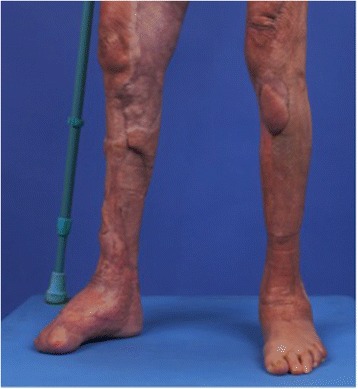
Appearance of a 55-year-old woman burn patient's legs at 6 months post-surgery, after the patient left the rehabilitation centre

A 55-year-old woman without relevant medical history was referred to our burn center with 35% total body surface area full thickness thermal burns (third degree) from flames on her lower limbs, hands and face (UBS 110, ABSI 9) (Fig. 1). The burns on her right ankle and foot and left knee seemed to be deeper (fourth degree). Initial surgical treatment occurred 2 h after the burns (Day 0) and included releasing incisions from knee to toes in both her lower limbs. The initial dressing was composed of silver sulfadiazine 1% and general resuscitation included fluid resuscitation and invasive monitoring. Then, three surgical procedures were required to excise the devitalized tissues, including muscles and tendons of both lower limbs. Thighs and upper third of her legs were covered with skin autografts. Tibia and fibula were exposed on the right limb. Toes were amputated through the metatarso-phalangeal joints. Ankle, mediotarsal and tarso-metatarsal capsules were burnt, some joints were open (Fig. 2) and vasculo-nervous pedicle was at high risk of exposure. Consequently, the lower extremity required covering with vascularized tissues. Local pedicled flap was not an option for the ankle and foot; therefore, we opted for a latissimus dorsi musculo-cutaneous flap with vertical skin paddle (4 × 20 cm) centered on the main perforator of the anterior branch of the thoraco-dorsal pedicle, identified with an echo-doppler. The flap was performed on day 27 post-burn. On the pre-operative CT-angiogram of the lower limb, we noticed that the anterior tibial artery was thrombosed just at the second third of the leg. The posterior tibial pedicle was preserved to ensure the vascularization of the remaining tissues of the foot. The thoraco-dorsal pedicle was end-to-end anastomosed to the anterior tibial artery. The anastomoses were difficult to perform because inflammatory tissues, full of oedema, surrounded the vessels. The skin paddle of the latissimus dorsi flap was originally intended for flap monitoring (Fig. 3). The free flap was a success, and 3 weeks later, the right limb was almost totally covered either with skin graft or with the flap. Nevertheless, the left patellar tendon was still exposed and needed to be covered with a flap (Fig. 4). The options discussed were to perform a pedicled flap, to perform a second free flap or to recycle tissues from the previous flap. We performed a vascular mapping of the flap with the use of an echo-doppler. The main pedicle of the flap was divided into two branches. The skin paddle was vascularized by this anterior branch from which a main perforator was identified. The choice between a musculo-cutaneous flap and a perforator flap was discussed. As a cross-leg had to be performed, the risks of rupture due to excessive tension or of thrombosis due to desiccation of the perforator were considered as too high. Consequently, a musculo-cutaneous cross-leg flap was harvested from the previous flap. The flap was partially raised from the right limb including the anterior branch of the thoraco-dorsal pedicle and a cross leg was performed to cover the left patellar tendon. Both legs were immobilized together using an external fixation (Hoffmann apparatus, Fig. 5). The donor site was covered with a skin graft. After a period of 3 weeks, the skin paddle was progressively cut from the previous flap after partial occlusion clamps were tested. One week later, the patient was discharged to a rehabilitation center for further treatment. The wounds were completely healed 9 weeks after the burns. At 6 months post-burns, the patient was able to walk and left the rehabilitation centre (Fig. 6).

## Discussion

In the majority of situations involving acute burns, a split thickness skin graft is the first option after debridement, as long as well-vascularized tissue remains. If the tissue left behind after excision is not suitable for a skin graft, local flaps are a second option. However, as the surrounding tissue may be involved in the zone of injury, local flaps are not always possible. As far as this patient was concerned, we concluded that a local flap, such as sural flap [10], could not cover the ankle and the foot because all the surrounding tissues were excised. Consequently, in such circumstances, free tissue transfer is the third option. Free flaps in burns are rarely indicated: in less than 2% of cases, including delayed post-burn reconstructions [1, 6].

We decided to perform a latissimus dorsi musculo-cutaneous flap. Whenever a large defect with dead space exists, a muscle flap is considered as a gold standard. The latissimus dorsi surface was large enough to cover the ankle and the foot. Moreover, the latissimus dorsi is a reliable flap with long pedicle and results in few sequelae at the donor site. A thoraco-dorsal artery perforator free flap was considered as a less safe alternative because of the hemodynamic instability of the patient and the possible use of vasopressive therapy. Indeed, the patient was treated with noradrenaline in order to maintain a stable blood pressure. This treatment is responsible for vasoconstriction of peripheral vessels including perforators. Consequently, we considered that a musculo-cutaneous flap, including more than one perforator would be safer and less sensitive to the effects of the treatment than a perforator flap based on a single perforator. The exposure of her left patellar tendon prompted us to perform a second flap. Local considerations were the same as the other limb for local flaps. An anterolateral thigh with a distal pedicle was discussed. However, the surface to cover were limited and donor sites for free tissue transfers are limited. Harvesting a second flap would have used another donor site for later reconstruction. Precisely in burns when some donor sites could be involved in the injuries.

The use of a previous redundant flap as a donor site is an interesting alternative in this situation [11–17]. Valauri et al. [12] reported the case of a patient who sustained bilateral below-knee amputations that were treated with skin grafts as initial coverage. A latissimus dorsi-free flap was later used as definitive coverage of one stump. During a subsequent operation, a portion of the same latissimus dorsi-free flap was harvested, again as a free flap, and transplanted to cover the contralateral stump. Thus, one latissimus dorsi-free flap was used twice as a free flap (free-flap free flap) to cover bilateral amputation stumps in sequential operations. Tan et al. [15] presented a case of a single free groin flap split in two to reconstruct two distant and separate defects sequentially. Chaput et al. [14] reported a case of puzzle flap in burns to cover the Achilles tendon after muscular retraction of the latissimus dorsi flap. The proposition advanced here was to combine the concepts of “puzzle flap” and “cross-leg flap” [18]. To avoid tension on the vascular pedicle and to improve venous drainage, we decided to perform a musculo-cutaneous flap including the perforator instead of a propeller flap, which could also have been done. The use of the musculo-cutaneous flap was also a solution to debulk the anterior face of the ankle and to avoid traction on the vessels during the cross leg phase.

## Conclusion

Sequential flap coverage may be considered in cases of extensive soft tissue defects, particularly in burns. With the advent of the concept of puzzle flap, reusing the monitoring part of a previous flap to cover another defect, in order to save donor sites for later reconstruction of contracted burn scars, is to be considered an interesting alternative. This innovative combination of a new concept (puzzle flap) and an old concept (cross leg) is an interesting example of the plasticity of flaps. Thanks to the great development of the identification of perforasomes, the surgeon would be able to recycle the skin paddle of a flap if perforators are found, which represents a second-line solution in the management of complex soft tissue defects.
